# Dr. Myra K. Merrick

**Published:** 1899-12

**Authors:** 


Dr. Myra K. Merrick, alleged to be the first woman who practised medicine in Ohio and one of the first who practised in the entire country, died recently at Cleveland (about November 11, 1899), at the age of 74 years.

